# Estrogen and Androgen Receptor Status in Uterosacral Ligaments of Women with Pelvic Organ Prolapse Stratified by the Pelvic Organ Prolapse Histology Quantification System

**DOI:** 10.1007/s43032-023-01283-z

**Published:** 2023-07-10

**Authors:** David J. Orlicky, E. Erin Smith, Rachel Bok, Marsha K. Guess, Lauren G. Rascoff, Jaime S. Arruda, Juana A. Hutchinson-Colas, Joshua Johnson, Kathleen A. Connell

**Affiliations:** 1 Department of Pathology, University of Colorado School of Medicine, Aurora, CO USA; 2 Department of Obstetrics and Gynecology, University of Colorado School of Medicine, Aurora, CO USA; 3 Robert Wood Johnson Medical School, Rutgers Health, New Brunswick, NJ USA

**Keywords:** Pelvic organ prolapse, Uterosacral ligament, Estrogen receptors, Androgen receptors

## Abstract

Menopause is a significant risk factor for pelvic organ prolapse (POP), suggesting that ovarian sex steroids play a major role in the etiology of the condition. POP results from failure of the uterine-cervix-vagina support structures, including the uterosacral ligament (USL). We previously identified consistent degenerative USL phenotypes that occur in POP and used their characteristics to develop a standardized POP Histologic Quantification System (POP-HQ). In this study, POP and matched control USL tissue was first segregated into the unique POP-HQ phenotypes, and specimens were then compared for estrogen receptor (ER) alpha (ERα), ERbeta (ERβ), the G-protein estrogen receptor (GPER), and androgen receptor (AR) content via immunohistochemical staining. ER and AR expression levels in the control USL tissues were indistinguishable from those observed in the POP-A phenotype, and partially overlapped with those of the POP-I phenotype. However, control-USL steroid receptor expression was statistically distinct from the POP-V phenotype. This difference was driven mainly by the increased expression of GPER and AR in smooth muscle, connective tissue, and endothelial cells, and increased expression of ERα in connective tissue. These findings support a multifactorial etiology for POP involving steroid signaling that contributes to altered smooth muscle, vasculature, and connective tissue content in the USL. Furthermore, these data support the concept that there are consistent and distinct degenerative processes that lead to POP and suggest that personalized approaches are needed that target specific cell and tissues in the pelvic floor to treat or prevent this complex condition.

**Supplementary Information:**

The online version contains supplementary material available at 10.1007/s43032-023-01283-z.

## Introduction

Pelvic organ prolapse (POP) is one of the complex, multifactorial conditions known as pelvic floor disorders (PFD). Nearly one-quarter of the women in the USA will experience a PFD with numbers anticipated to rise as the proportion of older individuals increases [1, 2]. In POP, the pelvic organs lose their support and descend downward into the vaginal canal [3]. Prevention and treatment of POP remains elusive due to a lack of understanding of its etiologies. The uterosacral-cardinal complex, including the uterosacral ligament (USL), provides apical support of the vagina and pelvic organs, and loss of this support is associated with advanced prolapse [4, 5].

The main known risk factors for POP include aging, parity with vaginal delivery, body-mass-index (BMI), and menopause [6, 7]. Since the hypoestrogenism from menopause corresponds to increased risk for prolapse, the premenopausal hormonal milieu may be protective. Cells within the support structures of the pelvic organs may be responding to estrogen which may play a key role in homeostasis of the extracellular matrix of the pelvic support system.

Estrogen exerts its actions through several receptors including nuclear and extranuclear estrogen receptor (ER) α (ERα) and ERβ, and the more recently identified G-protein-coupled estrogen receptor (GPER). The classical genomic actions of estrogen involve ligand activation of ERα and ERβ leading to dimerization and subsequent binding to ER response elements in the promoters of target genes that regulate gene expression [8–10]. In addition to genomic responses, estrogen can elicit rapid non-genomic signaling through the transmembrane G-protein-coupled estrogen receptor [11].

Multiple research techniques have been used in prior studies to examine the relationship between estrogen receptors and POP in USL tissue. However, available data are inconsistent between studies; messenger-RNA expression studies support increased [12] or decreased [13, 14] levels of ERα mRNA, along with increased estrogen receptor beta (ERβ) mRNA levels [12] in USL tissue from POP patients compared to matched controls. Other researchers have detected no change in the levels of ERβ mRNA but decreased levels of ERα at both the protein and mRNA levels in POP patients [16]. Two studies have reported that specific microRNAs (miRNAs) can regulate USL ERα or ERβ transcript levels [17, 18]. A genome-wide association study (GWAS) that evaluated genetic variants between POP and control patients identified a significantly overrepresented locus Rs3820282 that is predicted to alter a conserved binding site for ERα and ERβ, and to alter the estrogen-based regulation of WNT4 or adjacent genes [19].

Immunohistochemical staining (IHC) has been performed in a few studies evaluating the expression of ER and PR in USL. Mokrzycki et al. demonstrated positive ER staining only in the smooth muscle (SM) component in USL biopsies from 24 women without POP and 1 woman with POP; however, the specific ER type was not specified [20]. Dietrich et al. demonstrated the presence of ERα in the SM nuclei and ERβ in the endothelium of vessels in 13 women with and 13 women without prolapse undergoing hysterectomy [12]. Three other studies mention the presence of nuclear expression of ERα and ERβ in USL, but there is no description of which types of cells demonstrated positive staining [16–18].

While no studies have directly addressed androgen receptor status in the USL, some studies of pelvic floor support structures suggest that androgens may play a role in prolapse. For instance, castration induced a decrease in rat levator ani myocyte size that could be reversed by androgen treatment [21]. An increased cardinal ligament IHC-AR expression in women with POP [22], and an increase in vaginal wall and cardinal ligament AR expression have been reported in women with POP using IHC methodology, suggesting possible upregulation due to low circulating androgens [23]. Increased androgen metabolites have also been observed in women with POP [15]. Multiple review articles [24–26] suggest that androgens may have positive effects on the remodeling of female lower genital tissues. Supported by all of these prior studies, we have assessed androgen receptor levels, here, within USL biopsies.

In a prior study of USLs from women with POP, we demonstrated that semi-quantitative histopathological scoring followed by principal component analysis can separate these USLs into 3 main phenotypes that appear to correspond to different etiologies leading to the prolapse [27]. These histopathological phenotypes include increased inflammatory cells (POP-I), repair-associated changes in connective tissues (POP-A), and neointimal hyperplasia (NIH) related vascular changes (POP-V). This POP-V phenotype was found preferentially in post-menopausal women and suggests that hypoestrogenism may play a role in this older group of women with POP. Since the cell content in each of the POP phenotypes varies, it is important to characterize POP tissues prior to performing analyses to avoid conflicting results. This may explain the findings in previous studies of hormone receptors.

Since the expression and signaling mechanisms of ERα, ERβ, and GPER are complex and potentially exhibit redundant, independent, synergistic, and/or antagonistic actions, we chose to examine all three receptors and AR. In the present study, we hypothesized that USL ER and AR levels would differ between our histopathologically defined POP phenotypes. We utilized IHC to examine the ERα, ERβ, GPER, and AR content of multiple cell types within the USLs of the POP phenotypes as well as in the USLs of women that did not have POP. Interestingly, GPER and AR cell type expression were found to discriminate between POP phenotypes and controls as well as between the POP phenotypes, better than ERα and ERβ. Along with previously identified differences between the architecture, and cellular and extracellular features of POP ligaments, differences between steroid hormone receptors further support the segregation of POP USL tissue into specific phenotypes representing an additional step towards understanding POP etiology, and potentially, towards the personalization of POP treatment [28].

All abbreviations used in this manuscript are found in Table 1.

**Table 1:** List of abbreviations

Abbreviation	Meaning
POP	Pelvic organ prolapse
PFD	Pelvic floor disorder
USL	Uterosacral ligament
IHC	Immunohistochemical staining
POP-Q	Pelvic organ prolapse quantification system
Fibrillar Collagen	Trichrome stainable fibrillar collagen
Total Non-Vascular Smooth Muscle	Non-vascular, fascicular smooth muscle
SmFDO	Smooth muscle fiber dropout
Muscle Fiber Vesicles	Small vesicles found in some of the non-vascular smooth muscle cells
Adipose	Adipose found in the uterosacral ligament
PMN-Inflam	Neutrophil inflammation of the uterosacral ligament
PN-Inflam	Neutrophil inflammation surrounding the uterosacral ligament neural bundles
NIH	Neo-intimal hyperplasia
Vessel Quantity	Relative uterosacral ligament vascular quantity
POP-HQ	Pelvic organ prolapse histologic quantification system
POP-A	Prolapse adipose phenotype
POP-I	Prolapse inflammation phenotype
POP-V	Prolapse-vascular phenotype
ER	Estrogen receptor
ERa	Estrogen receptor alpha
ERb	Estrogen receptor beta
GPER	G-protein estrogen receptor
AR	Androgen receptor
ERa-SM	Estrogen receptor alpha in non-vascular, fascicular smooth muscle
ERa-CT	Estrogen receptor alpha in connective tissue
ERb-SM	Estrogen receptor beta in non-vascular, fascicular smooth muscle
ERb-ASM	Estrogen receptor beta in arterial tunica media smooth muscle
ERb-EC	Estrogen receptor beta in endothelial cells
ERb-CT	Estrogen receptor beta in connective tissue
GPER-SM	G-protein couple estrogen receptor in non-vascular, fascicular smooth muscle
GPER-ASM	G-protein couple estrogen receptor in arterial tunica media smooth muscle
GPER-EC	G-protein couple estrogen receptor in endothelial cells
GPER-CT	G-protein couple estrogen receptor in connective tissue
AR-SM	Androgen receptor in non-vascular, fascicular smooth muscle
AR-CT	Androgen receptor in connective tissue

## Materials and Methods

A Strengthening The Reporting of OBservational Studies in Epidemiology (STROBE) checklist was used to guide the experimental design and reporting of the demographic data [54].

### Specimen Collection

USL biopsies were obtained as previously described from women undergoing hysterectomy for benign gynecological conditions (i.e., abnormal uterine bleeding, symptomatic fibroid uterus) without POP (controls, CTL) and women with POP [27] with approval of the Colorado Multiple Institution Review Board (COMIRB #15–2245) and the Rutgers Institutional Review Board (#Pro20160001312). Written informed consent was obtained. Exclusion criteria included current pregnancy, previous POP or urinary incontinence surgery, or history of malignancy. Menopause was defined as no menses within the past year or previous oophorectomy. Using the Pelvic Organ Prolapse Quantification System (POP-Q), women with greater than or equal to stage II POP were assigned to the POP group and women with less than or equal to stage I POP were assigned to the control group [55]. Demographic data were collected for each subject. Demographic data for all subjects involved in this study have been uploaded to Figshare https://doi.org/10.6084/m9.figshare.21651494. During surgery, a 0.5-cm segment of each USL was biopsied within 1 cm of their cervical insertion and placed in neutral buffered formalin, paraffin embedded, sectioned, and trichrome stained. The full width of each USL section was examined and scored. A trained histopathologist (Orlicky) utilized the semi-quantitative histopathological POP-HQ scoring system [27], followed by principal component analysis using R packages “factoextra,” “fviz_pca,” “prcomp,” and “princomp” to separate the POP subjects into phenotypes as previously identified [27].

### Immunohistochemistry

The exact technique for IHC of each antigen is detailed in the Supplemental Materials Methodological details section. Data was collected as a percent positive of the cells for each cell type examined; an average of 300 cells in smooth muscle fascicles and connective tissue, and 125 cells within arterial smooth muscle fibers and endothelial cells; the last two tissue types are more limited in USL specimens. Our question was whether cells were or were not positive for each of the receptor types. As a key validation control, quantification of positive and negative cells was repeated in approximately one-quarter of all samples. Notably, and supporting reproducibility across the study, differences in values averaged 7% or less between counts of the same specimens.

### Data Analysis

POP phenotypes and control group composition were compared using R software for all statistical analyses, with required packages indicated where appropriate [29]. Welch’s unequal variances *t*-test was used when the phenotypes had unequal variances and were of unequal sample size. Analysis of variance with Tukey’s post hoc pairwise test was used when making multiple comparisons between continuous variables. Pearson’s Chi-squared test with Yates’ continuity correction was used for categorical variables (R package “RVAideMemoire”). For statistical comparisons of demographic data, *p* < 0.05 was considered significant. Principal component analysis (PCA) using R packages “factoextra,” “fviz_pca,” “prcomp,” and “princomp” was used to determine the influence of expression levels of each receptor type on POP-phenotype groupings. We have applied the “PCAtest” statistical analysis tool [56] to the PCA dataset.

## Results

This study included 15 control and 28 POP USL that were separated by the POP-HQ system into 8 POP-adipose (POP-A), 9 POP-inflammation (POP-I), and 11 POP-vascular (POP-V) phenotypes. Table 2 demonstrates the demographics of these POP-HQ phenotypes. POP-HQ data for all subjects involved in this study have been uploaded to Figshare, https://doi.org/10.6084/m9.figshare.21651494. Subjects in the POP-A phenotype differ from the controls in parity and menopause status, the POP-I phenotype differs in smoking incidence, and the POP-V phenotype differs in age, parity, and menopause. Table 3 indicates hormone use in each phenotype. The control group consisted of women with abnormal uterine bleeding and symptomatic fibroids and were more likely to be taking hormones, in particular oral progesterone. The POP-V group was more likely to be postmenopausal and had a higher incidence of using vaginal estrogen.

**Table 2:** Summary of the major demographics for the control and POP-phenotypes subjects

	Control	POP-A(dipose)	POP-I(nflammatory)	POP-V(ascular)
*N*	15	8	9	11
Feature	Mean ± SD	Mean ± SD	Mean ± SD	Mean ± SD
Age	46.2 ± 8.6^a^	50.8 ± 12.9^a^	53.7 ± 15.3^ab^	66.3 ± 8.3^b^
Vaginal deliveries	1.6 ± 1.4	3.0 ± 1.3	2.7 ± 1.3	2.7 ± 1.2
BMI	28.9 ± 6.1	30.8 ± 7.4	27.1 ± 4.6	29.3 ± 5.3
Menopause status	13 pre, 2 post	3 pre, 5 post	5 pre, 4 post	1 pre, 10 post
Smoking status	3 yes, 12 no	2 yes, 6 no	7 yes, 2 no	3 yes, 8 no

Average and standard deviations of the mean are included for the main demographic features for each phenotype of subjects. The *N* of each group is indicated. Mean values that significantly differ from one another are denoted by letters (a,b); *p* < 0.05 as determined by ANOVA followed by Tukey’s post hoc pairwise test

**Table 3:** Hormone use in controls and POP-HQ groups

	CONTROL	POP-A(dipose)	POP-I(nflammatory)	POP-V(ascular)
*N*	15	8	9	11
Hormone				
No hormone use	9 (60%)	8 (100%)	6 (66.7%)	4 (36.4%)
Oral estrogen	0 (0%)	0 (0%)	0 (0%)	0 (0%)
Oral progesterone	4 (26.6%)	0 (0%)	0 (0%)	0 (0%)
Oral contraceptive Pills	1 (6.7%)	0 (0%)	1 (11.1%)	0 (0%)
IUD (progesterone)	1 (6.7%)	0 (0%)	1 (11.1%)	0 (0%)
Vaginal estrogen	0 (0%)	0 (0%)	1 (11.1%)	7 (63.6%)
Other- transdermal estrogen, transdermal progesterone, oral and transdermal testosterone	0 (0%)	0 (0%)	0 (0%)	0 (0%)

A summary of the hormone replacement therapy for the subjects in these phenotypes is included

Table 4 shows the averages, standard deviations, and statistical comparisons for the histopathological scoring features present in the POP-HQ defined, POP-phenotype groups of subjects used in this study. The POP-A phenotype had less smooth muscle and more muscle fiber dropout, the POP-I phenotype has more inflammation, and the POP-V phenotype has thicker tunica intima, as observed previously [27].

**Table 4:** Summary of the POP-HQ histopathological scoring for the control and POP-phenotypes USLs

	Control	POP-A(dipose)	POP-I(nflammatory)	POP-V(ascular)
*N*	15	8	9	11
Feature	Mean ± SD	Mean ± SD	Mean ± SD	Mean ± SD
Fibrillar collagen	53.8 ± 17.0	67.1 ± 14.0	54.9 ± 13.9	53.2 ± 26.0
Total non-vascular smooth muscle	2.4 ± 0.5^a^	1.5 ± 0.6^a^	2.0 ± 0.7	2.0 ± 0.7
SmFDO	0.8 ± 0.5^a^	1.4 ± 0.3^a^	1.3 ± 0.5	0.9 ± 0.5
Muscle fiber vesicles	0.0 ± 0.1	0.0 ± 0.0	0.0 ± 0.0	0.2 ± 0.5
Adipose	0.4 ± 0.7	0.8 ± 0.5	0.7 ± 0.6	0.3 ± 0.3
PMN-Inflam	0.4 ± 0.2^b^	0.9 ± 0.8^ab^	1.9 ± 0.7^b*^	0.1 ± 0.1^ab^
PN-Inflam	0.1 ± 0.1^a^	0.3 ± 0.7	0.6 ± 0.8^ab^	0.0 ± 0.0^b^
NIH	0.4 ± 0.8^a^	0.0 ± 0.0^a^	0.0 ± 0.0^a^	2.3 ± 0.6^a*^
Vessel quantity	1.1 ± 0.8	0.9 ± 0.5	1.2 ± 0.8	1.7 ± 0.6

Average and standard deviations of the mean are included for the POP-HQ histopathological scoring features for each phenotype of subjects. The *N* of each group is indicated. Statistical comparisons of POP-HQ phenotypes’ histopathological scoring data were performed using R software with required packages indicated where appropriate [29]. Welch’s unequal variances* t*-test was used when the phenotypes had unequal variances and were of unequal sample size. Analysis of variance with Tukey’s post hoc pairwise test was used when making multiple comparisons between continuous variables and Pearson’s Chi-squared test with Yates’ continuity correction for categorical variables (R package “RVAideMemoire”). Mean values that significantly differ from one another are denoted by letters (a, b, c); *p* < 0.05 as determined by ANOVA followed by Tukey’s post hoc pairwise test. Asterisks indicate significance where *p* < 0.005 between indicated group and all other groups

**Fig. 1:**
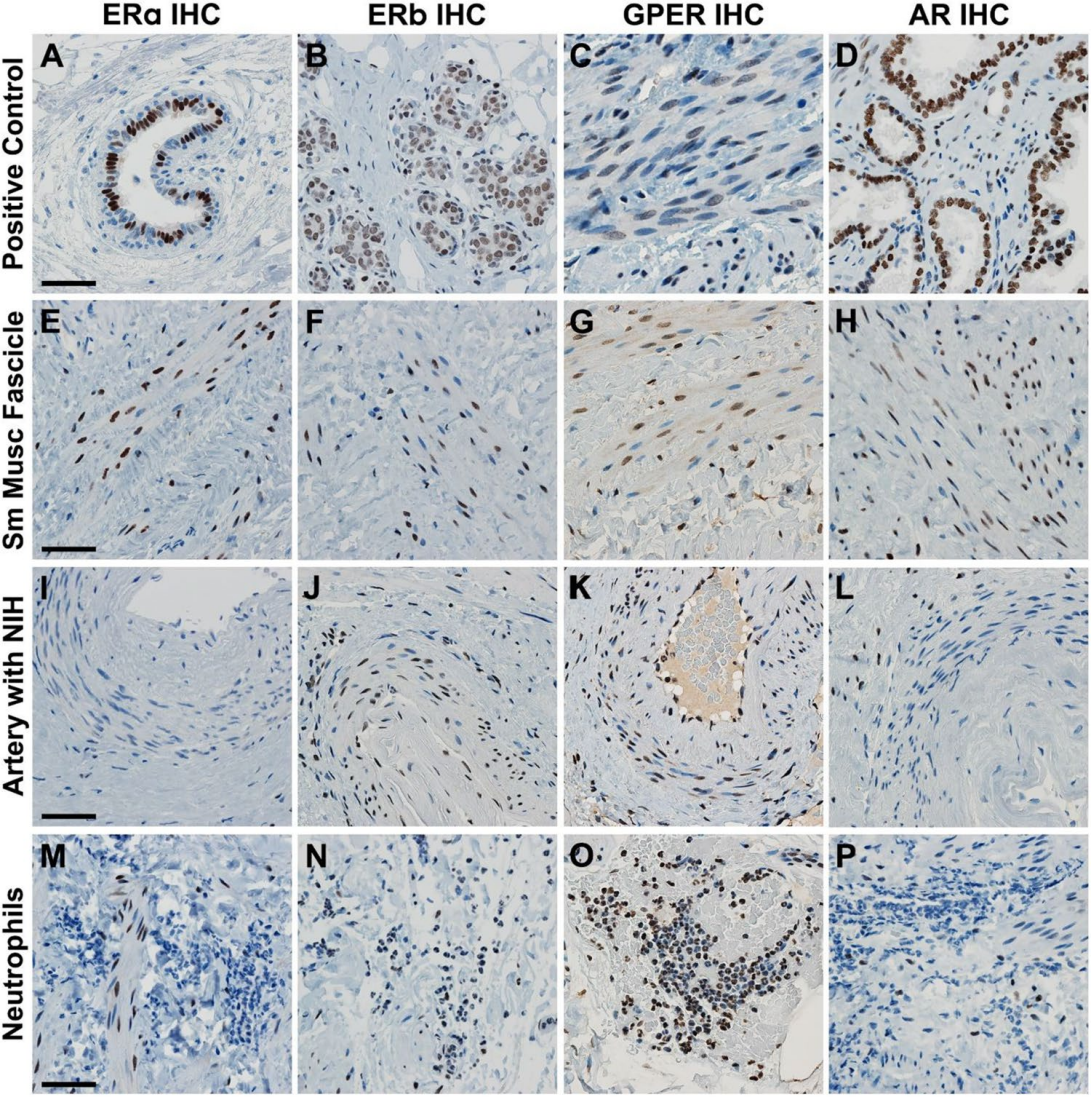
Immunohistochemical staining for ERα, ERβ, GPER, and AR. IHC staining for each of the receptors is demonstrated on the antibody manufacturer’s suggested control (images **A**–**D**), USL smooth muscle fascicles (**E**–**H**), arteries with NIH (**I–L**), and on PMN inflamed tissue (**M–P**). A more extensive example figure for each IHC is provided in the supplementary Figs. 1a–d. All images shown were shot at the same magnification (× 400). All size bars are 50 µm

**Fig. 2:**
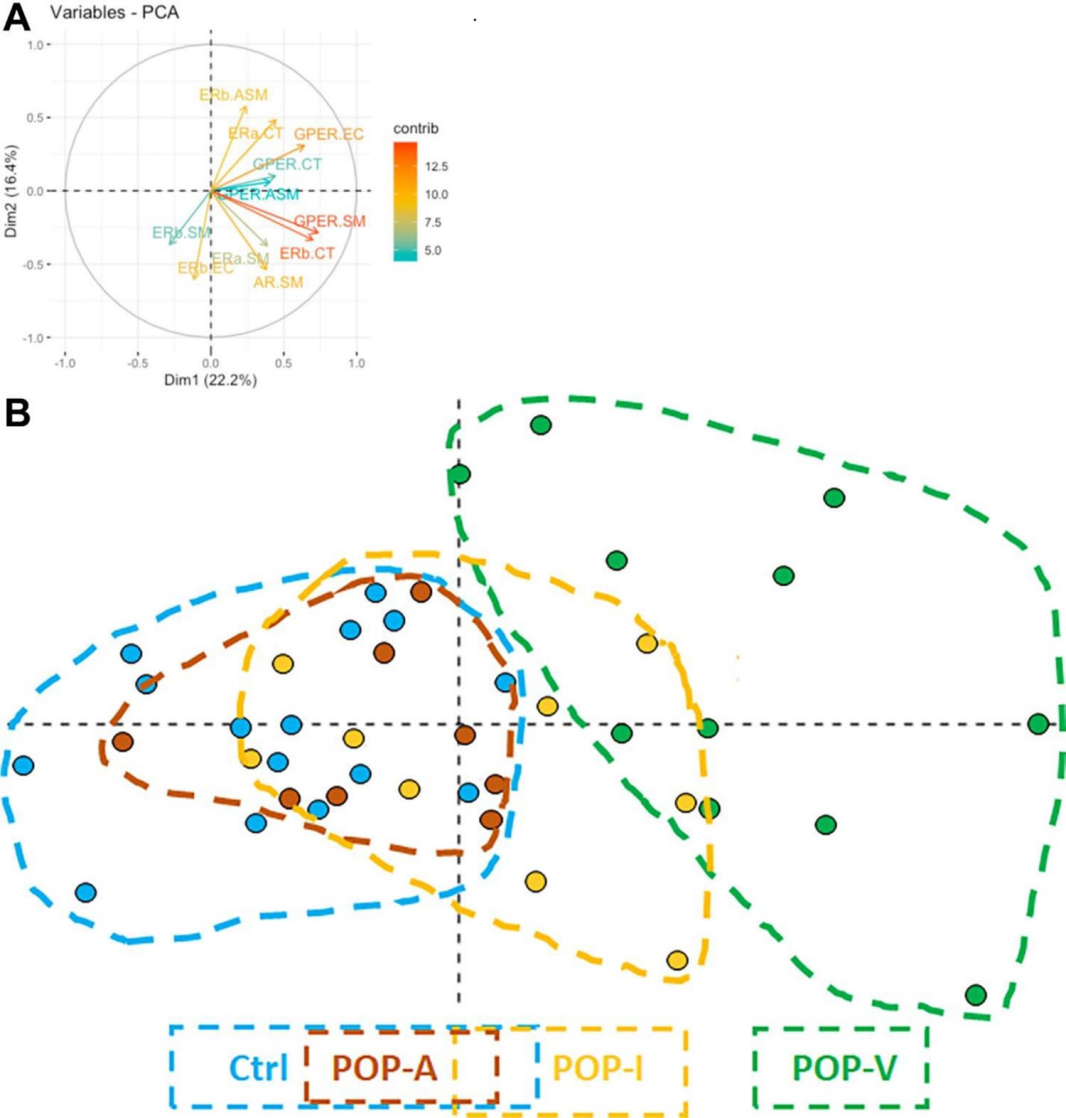
PCA diagram of receptor IHC data. Portion **A** shows the vectors for dispersion including strength and direction. Portion **B** shows the PCA distribution of all receptor IHC staining quantification data for all the subjects. Dashed outlines surround the various POP-phenotypes. Control subjects (blue) completely overlapped the POP-A subjects (brown). Control and POP-A subjects largely overlapped the POP-I phenotype subjects (tan). In contrast, the POP-V subjects (green) are almost completely separated from the three other phenotypes

Figure 1 demonstrates IHC staining for each of the 4 steroid receptor types analyzed; it includes the tissue utilized by the antibody’s commercial source as a positive control, followed by staining of the USL smooth muscle bundles, arteries with NIH, and neutrophil accumulations in connective tissue. Supplementary Figs. 1A–D demonstrate more examples from the USL IHC for each of these steroid receptors. The contrast in receptor localizations bears further mention. Table 5 summarizes the USL cell types that stained positively for the presence of each of the indicated receptors. Interestingly, all four receptor subtypes were found expressed in a percentage of smooth muscle fascicle myocytes, yet only ERβ and GPER were expressed in arterial smooth muscle myocytes. Adipocytes were positive for ERβ, GPER, and AR proteins. At least some connective tissue cells were positive for all 4 receptors. Neutrophils were positive for ERβ and GPER, as were tunica intima cells and endothelial cells. Quantification of the percentage of positive cells is included for smooth muscle fascicle myocytes, endothelial cells, connective tissue, and vascular smooth muscle myocytes. Although neural bundles were found in all USLs, their presence was highly variable in quantity; and thus, we could not be sure at least 125 Schwann cells would be present in all specimens, so quantification of the presence of their steroid receptors was not performed. Similarly, adipocytes and neutrophils were found only in the POP-A and POP-I USL phenotypes; therefore, their steroid receptor content was not quantified. Table 6 summarizes quantification of this data and their statistical comparisons between the controls and each USL-phenotype. All scoring for sex steroid levels for all subjects have been uploaded to Figshare, https://doi.org/10.6084/m9.figshare.21651494. Figure 2 is a PCA plot of all steroid receptor data, with separation of those plotted samples for the CTL and POP-phenotypes (dashed outlines), and a vector diagram indicating the relative strength and direction that each receptor-cell type contributed to the separation. The POP-A phenotype overlaps that of the control phenotypes entirely, as both trended toward higher expression of ERβ in the endothelial cells and connective tissue compared to the POP-I and POP-V phenotypes; however, there is partial overlap with the POP-I phenotype. The expression of the receptors in the POP-I group tended to be like the CTL/POP-A groups, or between these and the POP-V group. The POP-V phenotype separates from the others, principally due to connective tissue AR expression and fascicle smooth muscle myocyte GPER expression. We have applied the “PCAtest” statistical analysis tool [55] to the PCA dataset. PCAtest output tests “(1) the hypothesis that there is more correlational structure among the observed variables than expected by random chance, (2) the statistical significance of each PC, and (3) the contribution of each observed variable to each significant PC.” That test detected significance in our PCA approach and test results are provided as follows:

- Test of PCA significance: 12 variables, 43 observations, 100 bootstrap replicates, 100 random permutations
- Empirical Psi = 7.1954, Max null Psi = 4.5593, Min null Psi = 1.9514, *p*-value = 0
- Empirical Phi = 0.2335, Max null Phi = 0.1858, Min null Phi = 0.1216, *p*-value = 0

**Table 5:** IHC results for each USL cell type

Cell type	ERα	ERβ	GPER	AR
Positive control #1	Mammary gland	Mammary gland	Endometrium	Prostate
Positive control #2	None	Brain	None	None
Smooth muscle fascicle	Yes	Yes	Yes	Yes
Adipocytes	No	Yes	Yes	Yes
Neural bundle Schwann Cells	No	Yes	Yes	No
Endothelial cells	No	Yes	Yes	No
Connective tissue	Yes	Yes	Yes	Yes
Arterial smooth muscle	No	Yes	Yes	No
Neutrophils	No	Yes	Yes	No
Foamy macrophages	No	Yes	Yes	No

A summary of the IHC staining for each of the steroid receptor subtypes is shown here; the demonstration of each of these results is supplied in the supplementary Fig. 1a–d. The main IHC staining results are demonstrated in Fig. 1

**Table 6:** Summary for the steroid receptor IHC staining data for the POP and control USL phenotypes

		Control	POP-A(dipose)	POP-I(nflammatory)	POP-V(ascular)
	*N*	15	8	9	11
Receptor	Cell type	Mean ± SD	Mean ± SD	Mean ± SD	Mean ± SD
ER alpha	Smooth muscle	43.3 ± 13.9	58.4 ± 4.3	53.8 ± 10.2	55.5 ± 17.5
	Connective tissue	19.9 ± 11.7^a^	20.1 ± 5.8	17.0 ± 6.5^b^	33.1 ± 14.9^ab^
ER beta	Smooth muscle	55.4 ± 3.6	55.5 ± 4.8	50.8 ± 5.0	51.6 ± 4.5
	Arterial smooth muscle	56.3 ± 4.6	62.0 ± 7.8	57.7 ± 4.9	62.9 ± 8.3
	Endothelial cells	68.7 ± 4.7	65.8 ± 8.0	70.3 ± 3.3^a^	63.4 ± 6.0^a^
	Connective tissue	34.5 ± 4.2^a^	38.1 ± 3.1	38.7 ± 6.2	40.7 ± 6.7^a^
GPER	Smooth muscle	54.7 ± 5.6^a^	54.9 ± 4.5^b^	60.4 ± 6.3	66.4 ± 8.9^ab^
	Arterial smooth muscle	64.0 ± 5.7	62.1 ± 3.6	62.0 ± 3.3	67.9 ± 8.0
	Endothelial cells	73.9 ± 6.9^a^	66.0 ± 5.1^a*^	74.1 ± 6.2^a^	78.0 ± 5.9^a^
	Connective tissue	63.8 ± 5.5^a^	66.4 ± 5.0^a^	63.6 ± 4.2^a^	74.7 ± 8.5^a*^
AR	Smooth muscle	35.7 ± 7.3	36.5 ± 8.5	36.8 ± 9.3	42.8 ± 10.0
	Connective tissue	17.4 ± 4.1^a^	18.1 ± 3.7^b^	25.8 ± 9.5	34.0 ± 11.3^ab^

Average and standard deviations of the mean are included for the results of each steroid receptor IHC for each phenotype of subjects. The *N* of each group is indicated. Statistical comparisons of the steroid receptor IHC data for all phenotypes of subjects were performed using R software with required packages indicated where appropriate [29]. Welch’s unequal variances *t*-test was used when the phenotypes had unequal variances and were of unequal sample size. Analysis of variance with Tukey’s post hoc pairwise test was used when making multiple comparisons between continuous variables and Pearson’s Chi-squared test with Yates’ continuity correction for categorical variables (R package “RVAideMemoire”). Mean values that significantly differ from one another are denoted by letters (a, b); *p* < 0.05 as determined by ANOVA followed by Tukey’s post hoc pairwise test. (*) indicate significance where *p*<0.05 between indicated group and all other groups.

- PC 1 is significant and accounts for 25.4% (95%-CI: 21.2–31.8) of the total variation

Variables 6 (ERbeta connective tissue), 7 (GPER smooth muscle), 9 (GPER endothelial cells), and 12 (AR connective tissue) have significant loadings on PC 1. PCA test indicates that these results are significant assuming a *p* = 0.05.

**Fig. 3:**
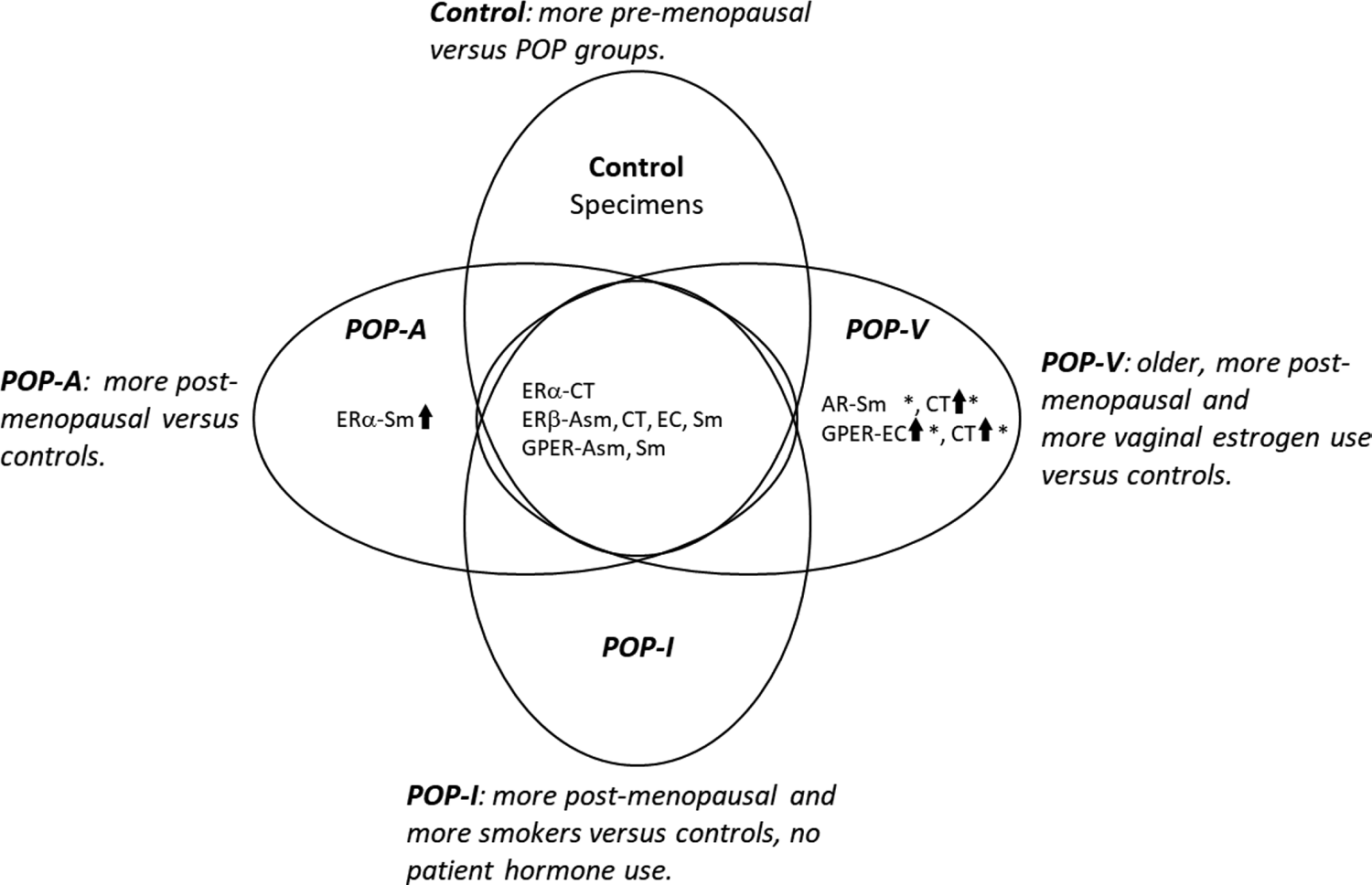
Data summary. Differences in steroid receptor content were detected between USL specimens from control (non-POP) and POP patients. Significant differences in the percentage of immunostaining-positive cells (by indicated cell type(s)) are indicated by non-overlap in the Venn diagram. Up arrows indicate higher fractions of positive cells than controls. Asterisks indicate significant steroid receptor expression differences when only post-menopausal data are included in the analysis (post-menopausal POP specimens were combined into a single group, Welch’s *t* test). POP-A, POP-Adipose; POP-I, POP-Inflammatory; POP-V, Pop-vascular phenotypes; Asm, arterial smooth muscle; CT, connective tissue; EC, endothelial cells; Sm, smooth muscle

A summary graphic (Fig. 3) is provided that includes the major demographic features, histological alterations observed  [27] and the major sex-steroid receptor findings.

## Discussion

The use of steroid hormone therapy to prevent or even reverse the effects of tissue senescence in women has been explored extensively. Estrogen or testosterone, or both, can prevent decreases in collagen content associated with aging in post-menopausal women [30], and treatment of post-menopausal women with estrogen can increase skin collagen content [31]. These observations, together with the increase in POP after menopause when sex steroids are decreasing, have prompted studies to evaluate the effects of various steroids on prolapse. Although estrogen is effective at restoring biochemical properties of pelvic connective tissues in ovariectomized young rats, it does not do so in middle-aged rats [32]. Furthermore, estrogen delivery coupled with use of various progestogen regimens has been associated with an elevated risk of uterine prolapse in postmenopausal women [33]. Multiple reports claim estrogen inhibits the proliferation of vascular [34–36] and vaginal smooth muscle [37]. Estrogen also stimulates pelvic collagen degradation accompanied by minor amounts of collagen synthesis [38] and suppresses proliferation of fibroblasts derived from cardinal ligaments [39]. Thus, the use of estrogen to treat prolapsed tissues is complex and unresolved [40].

Previous reports have indicated a positive effect of androgens on the remodeling of female lower genital tissues [reviewed in [24–26]]. However, many of these reports detail effects upon skeletal muscle rather than smooth muscle [24–26, 41–43] and therefore would not be expected to affect the USL. Although androgen treatment has been reported to reverse a castration-induced decrease in levator ani myocyte size [21], others have concluded that any hormone therapy effect on pelvic organ support was likely to be small [44].

To varying degrees, all previous studies designed to elucidate a potential sex-steroid and prolapse association have failed to account for the possibility of multiple prolapse etiologies, or that multiple types of receptors exist for some steroids, and that these receptors may exist on multiple cell types. Our highly individualized approach accounted for the multiple etiologies of POP by using the POP-HQ histopathological scoring system and included a wider array of the sex steroid receptors and their specific cells of expression through IHC histological assessment. The use of principal component analysis allowed changes in all receptor-cell type subsets to be considered simultaneously.

Following this individualized approach, the USL-tissue steroid receptor status of the POP-A did not differ from control USL-tissue. USL-tissue from the POP-I phenotype differed from the control group only in the quantity of a single steroid receptor. However, the POP-V phenotype separated away from the others following our cell type-sex steroid receptor quantification, and the steroid receptor differences driving this separation were GPER and AR expression with smaller contributions from age and menopause-dependent increased ERα and ERβ expression. The use of IHC confirmed both that several tissues/cell types within the USL possess steroid receptors and that not all of them change uniformly. Prior treatment of the USL as a single entity by homogenizing it, or by assessing only a single cell type (smooth muscle or fibroblast) within it following IHC, has dismissed the complexity of the tissue.

The steroid receptor differences identified here may be of particular importance to both the POP-V and POP-I phenotype etiologies. Relevant to the POP-V phenotype, estrogen has been reported to possess atheroprotective effects in both ERα-KO and ERβ-KO animals (implicating GPER), possibly through a mechanism that includes accelerated endothelial cell growth [45]. We observed GPER in cells of the neointima of POP-V USL-tissue, in arterial smooth muscle myocytes, in endothelial cells, and in neutrophils. Relevant to the POP-I phenotype, estrogen increases macrophage activity through GPER [46] and increases a proinflammatory reaction with cytokine production in neutrophils through GPER [47]. Our POP-I phenotype has extensive neutrophil involvement.

Lastly, our prolapsed POP-A phenotype displays decreased smooth muscle content, and increased collagen and adipose tissue compared to control USLs, yet tissues in this phenotype have similar sex steroid receptor content to that found in control subjects, thus reiterating that steroid receptors are not the answers to all types of POP and its failing tissues.

### Strengths and Weaknesses

The major strengths of our study are, first, utilizing better-defined study groups through identification of POP phenotypes using of the POP-HQ scoring system, and second, the examination of ERα, ERβ, GPER, and AR altogether in the same USL-tissues of POP and control subjects. Together, these results reinforce the hypothesis that multiple degenerative processes are leading to POP. The identification of patient clinical phenotypes for each of the POP-phenotypes may lead to actionable strategies based upon use of sex steroids or antagonists thereof either alone or in combination. Agonists and antagonists for all 4 of these steroid receptors now exist [41–43, 48–50]. We hypothesize that women with different POP-phenotype-steroid receptor signatures will have differential risks for complications or recurrence; thus, knowledge from studies like this one is essential for personalized medicine.

There are limitations to our study. The women in our control group are not representative of the general population since they were undergoing hysterectomy for abnormal uterine bleeding and symptomatic fibroids. Four control subjects were taking oral progesterone and one was taking oral contraceptive pills. Also, a much larger percentage of POP-V patients were using vaginal estrogen than the other groups. A few women in both groups had a progesterone-eluting IUD, and the effects of this on the USL are also unknown. However, we found expression of all the receptors in all studied cell types in both the control and POP phenotype groups regardless of subgroup or hormone use. Lastly, the POP-V subjects were older than the control or POP-A subjects. Future, larger studies are necessary to achieve adequate sample sizes to control for hormone use and age, and to determine their effects on sex steroid receptors.

We have stained for and quantified steroid receptors with the understanding that the presence or absence of a receptor does not necessarily indicate a functional consequence. We did not measure the levels of the sex steroids, and the presence of the steroid is as important as the receptor. In addition, the impact of steroid hormone supplementation (as in hormone replacement therapy, HRT) in postmenopausal women has not been shown to slow the progression of POP [51–53]. However, the large studies that tested for any impact of HRT upon POP progression did not consider that the support structures of individual women might differ from one another, as shown in the POP-HQ USL categories [27] and in their differential steroid hormone receptor expression shown here. It remains possible that subsets of women with particular USL phenotypes might benefit from HRT given their receptor profile. Lastly, the USL only provides one of the three levels of support for the pelvic organs and is not expected to be solely responsible for the development of POP. Undoubtedly, failure of other levels of support also contribute to prolapse, and a comprehensive study of expression of sex steroid receptors throughout the pelvic support system is warranted.

In conclusion, we have utilized an IHC approach to examine the ERα, ERβ, GPER, and AR content of various cell types in the USL-tissues of women with prolapse from histopathologically distinct POP phenotypes. Increased GPER and AR levels were major discriminators for separating the POP-phenotypes patients from controls while ERα and ERβ were minor discriminators. Levels of estrogen and androgen receptors and their cell type specificities separate POP-phenotypes into those that completely overlap with, that partially overlap with, or that separate completely from control USL-tissues at *p*-values of < 0.05. A summary of the important demographics, histological features, and sex-steroid receptor expression is provided (Fig. 3). Analysis of these data suggests that prior disparities in reported steroid receptor expression in the USL may stem from categorical differences in USL content (e.g., the POP-HQ phenotypes) that themselves may modify POP progression or steroid hormone responsiveness.

### Supplementary Information

Below is the link to the electronic supplementary material.

Supplementary file1 (DOCX 9585 KB)

## Data Availability

Complete data used to prepare this manuscript submission have been uploaded to Figshare. No patient identifying information is included. Demographic and histological quantitative and categorical information are provided in an Excel database file (link: https://doi.org/10.6084/m9.figshare.21651494).
