# Supplementary material for: Estrogen and Androgen Receptor Status in Uterosacral Ligaments of Women with Pelvic Organ Prolapse Stratified by the Pelvic Organ Prolapse Histology Quantification System
Source: Reprod Sci. 2023 Jul 10;30(12):3495–506. doi: 10.1007/s43032-023-01283-z (PMC10692001; doi:10.1007/s43032-023-01283-z)
Supplement: Supplementary file 1 — Supplementary file1 (DOCX 9585 KB) [file 43032_2023_1283_MOESM1_ESM.docx]

Supplemental information

*Supplemental Methodological details for the IHC*

Four to five micron thick paraffin sections were prepared for immunodetection of estrogen receptor alpha (Neomarkers/Thermo Scientific, Waltham, MA; rabbit monoclonal SP6; 1:500), estrogen receptor beta (BioRad, Hercules, CA; mouse monoclonal PPG5/10; 1:100), androgen receptor (Cell Marque/MilliporeSigma, Rocklin, CA; rabbit monoclonal SP107; 1:100), and GPER (Antibodies Online/Bioss, Limerick, PA; rabbit polyclonal; 1:1000). Antigens for estrogen receptor alpha and androgen receptor were revealed in BORG solution (Biocare Medical, Concord, CA; pH 9.5) and antigens for estrogen receptor beta and GPER were revealed in sodium citrate buffer (10 mM, pH 6.0 + 0.1% Tween 20), respectively, for 10 minutes at 110°C (NxGen Decloaker, Biocare). The slides were cooled for 10 minutes at room temperature after pressure release. Primary antibodies were diluted in Reaction Buffer (Ventana Medical Systems, Roche Diagnostics, Indianapolis, IN) spiked with 1% bovine serum albumin. Automated immunodetection of estrogen receptor alpha, androgen receptor and GPER was performed on the Benchmark XT autostainer (Ventana, Roche, Indianapolis, IN) with primary antibody incubation for 32 minutes at 37℃ using the UltraView DAB polymer detection (Ventana). Manual immunodetection of estrogen receptor beta was performed at ambient temperature in a humidified chamber using the Mouse ImmPress polymer detection system (Vector Laboratories, Newark, CA; cat# MP-7402). Endogenous peroxidase was quenched with 3% hydrogen peroxide for 10 minutes. This was followed by a 2.5% normal horse serum protein block for 1 hour provided in the Mouse ImmPress kit. Primary antibody PPG5/10 was applied and incubated for 1 hour followed by tris-buffered saline rinses (5 min each X 3). Mouse ImmPress polymer was applied and incubated for 30 minutes followed by tris buffered saline rinses (5 min each X 3). DAB was used to visualize the antigen-antibody complexes for 5 minutes. All immunostained sections were counterstained in Harris hematoxylin for 2 minutes, blued in 1% ammonium hydroxide, dehydrated in graded alcohols, cleared in xylene and coverglass mounted using synthetic resin.

Negative controls to confirm the specificity of the immunostaining included omission of the primary antibody incubation step in the IHC protocol, substitution of the primary antibody diluent.


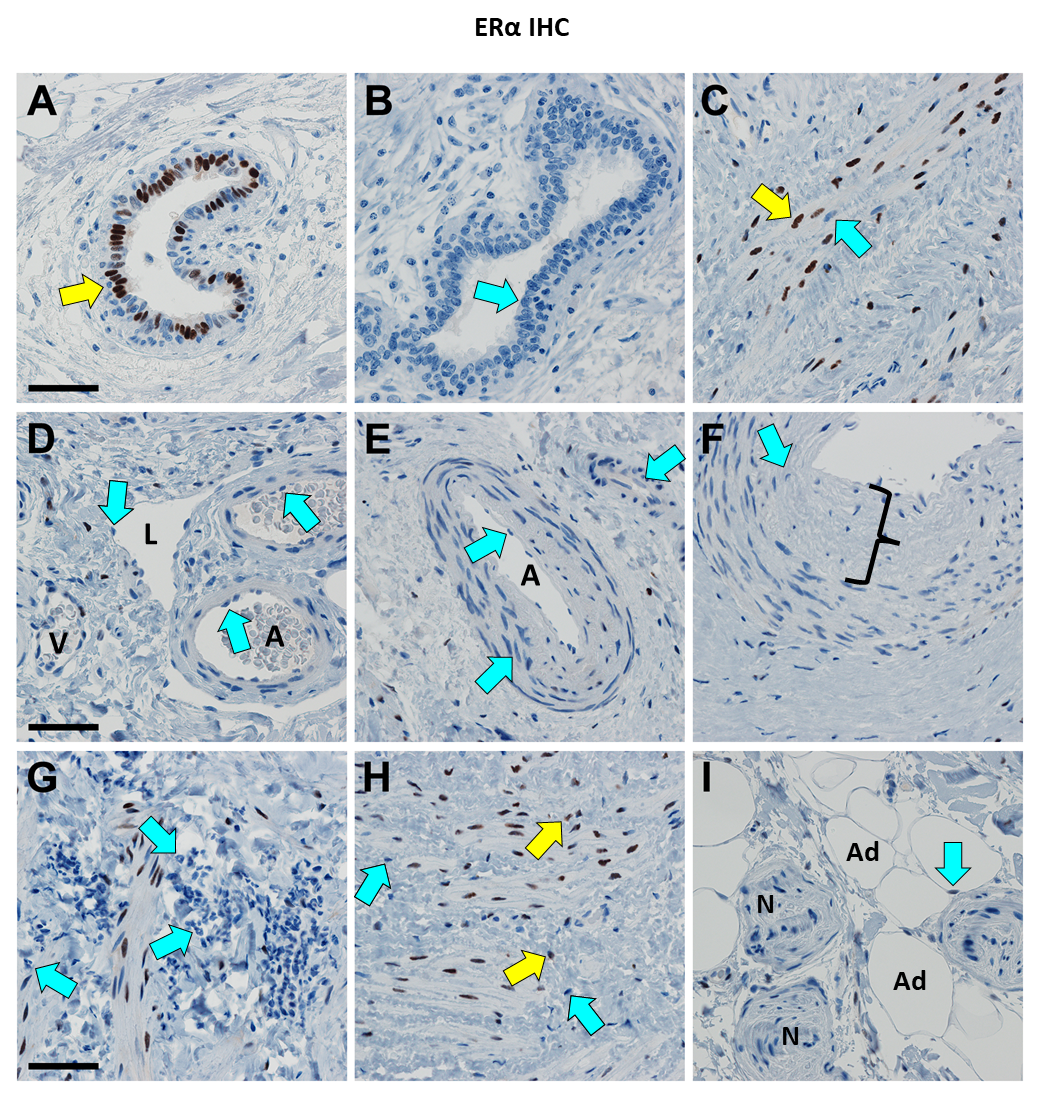


*Supplemental Fig 1a Estrogen Receptor alpha immunohistochemistry examples* ER**α** IHC is shown for A) positive control, mammary gland, B) mammary gland staining without use of the primary antibody (negative control), C) USL smooth muscle fascicle, D) arteriole (A), vein (V), and lymphatic (L) showing endothelial cells, E) large artery with normal 1 layer tunica intima showing arterial smooth muscle and endothelial cells, F) large artery with NIH (bracket) showing tunica media, neointima cells and endothelial cells, G) neutrophils (yellow arrows) between smooth muscle fascicles, H) IHC positive cells in the connective tissue between the smooth muscle fascicles, and I) neural bundles (N) and adipocytes (Ad). IHC positive cells are indicated by yellow arrows, IHC negative cells are indicated by light blue arrows. Size bars are 50 microns.


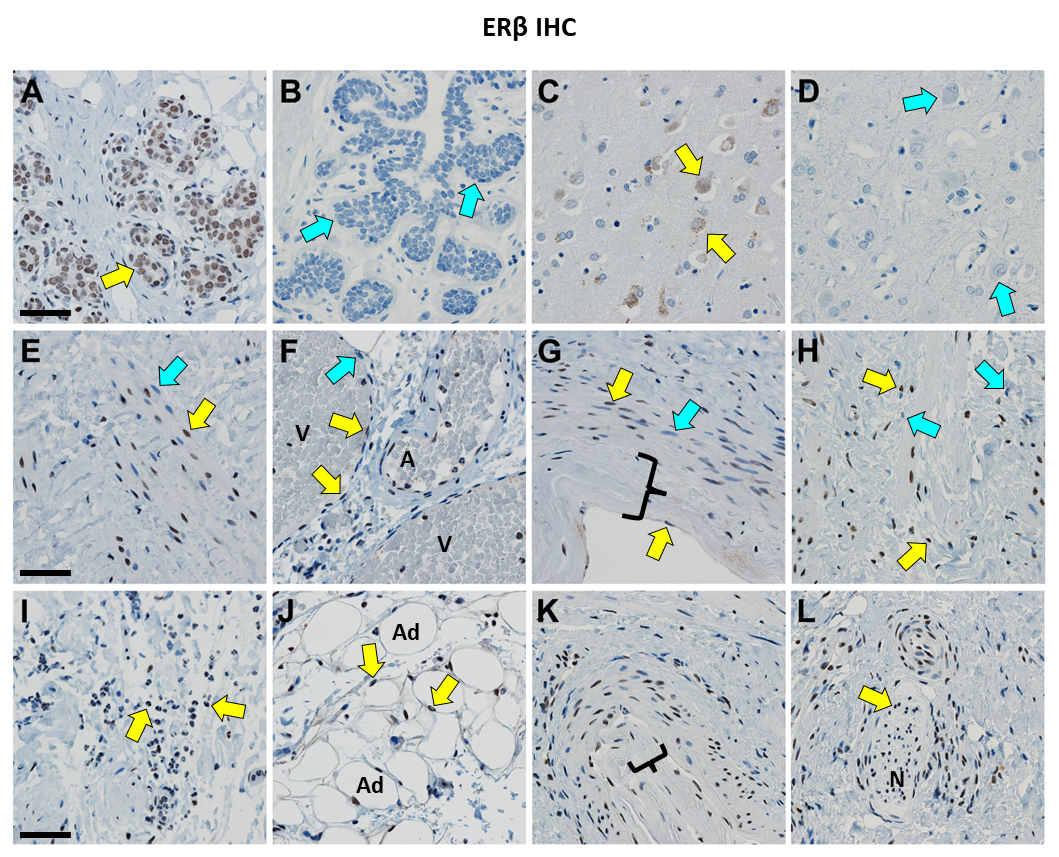


*Supplemental Fig 1b Estrogen Receptor beta immunohistochemistry examples* ERβ IHC is shown for A) positive control, mammary gland, B) mammary gland staining without use of the primary antibody (negative control), C) positive control, brain, neural cells, D) brain neural cells without use of the primary antibody (negative control), E) USL smooth muscle fascicle, F) arteriole (A) and veins filled with red blood cells (V) showing endothelial cells, G) large artery with NIH (bracket) showing tunica media, neointima cells and endothelial cells, H) IHC positive cells in the connective tissue between the smooth muscle fascicles, I) neutrophils between smooth muscle fascicles, J) adipocytes (Ad), K) large artery with neointima hyperplasia (bracket) and revascularization, and L) a neural bundle (N). IHC positive cells are indicated by yellow arrows, IHC negative cells are indicated by light blue arrows. Size bars are 50 microns.


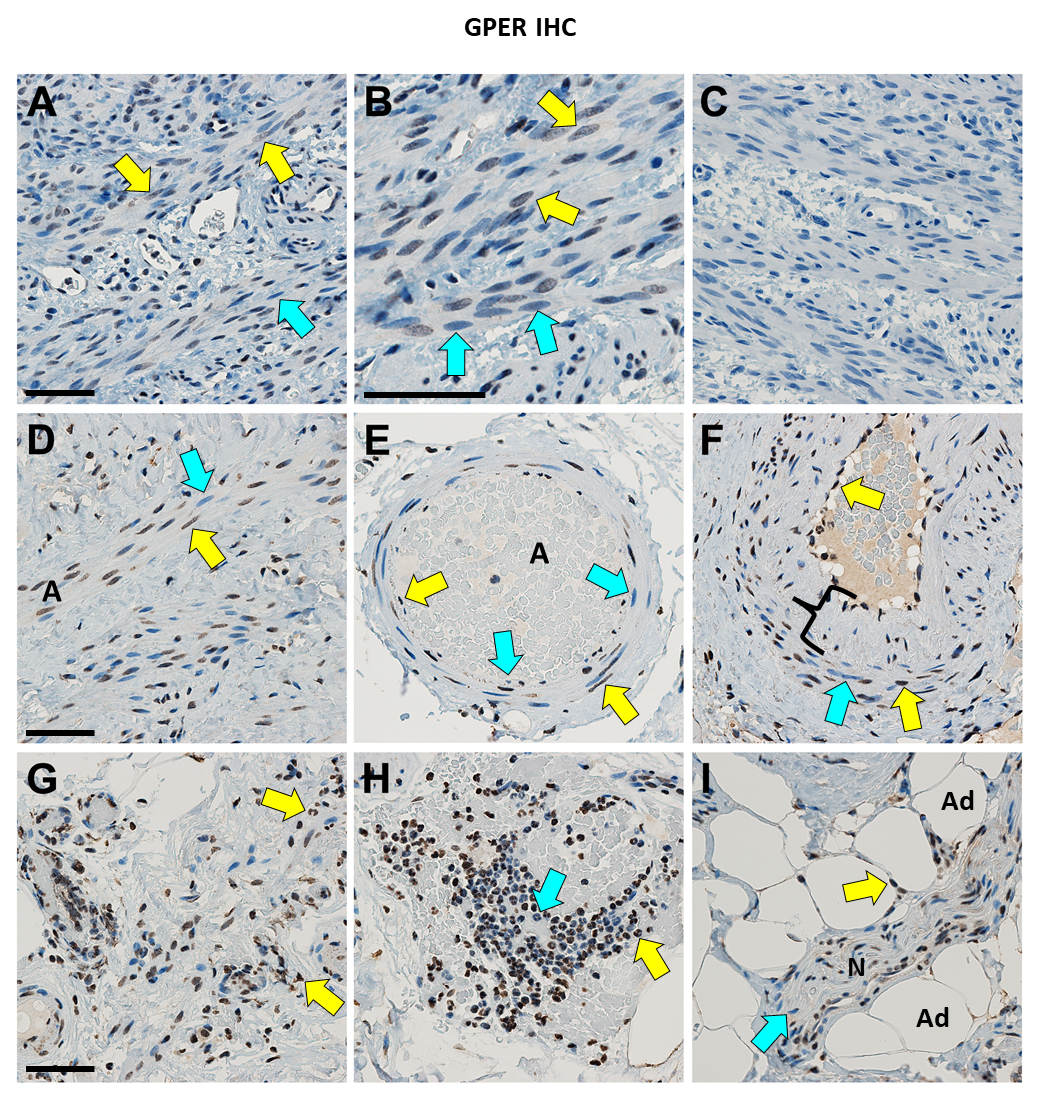


*Supplemental Fig 1c G-Protein Estrogen Receptor immunohistochemistry examples* GPER IHC is shown for A) positive control endometrium, B) higher magnification positive control endometrium, C) endometrium mammary gland staining without use of the primary antibody (negative control), D) USL smooth muscle fascicle, E) artery (A) with 1 layer of tunica intima showing endothelial cells, F) large artery with NIH (bracket) showing tunica media, neointima cells and endothelial cells, G) neutrophils in connective tissue, H) neutrophils, some IHC positive (yellow arrows) and some IHC negative (blue arrows) near smooth muscle fascicles, and I) neural bundles (N) and adipocytes (Ad). IHC positive cells are indicated by yellow arrows, IHC negative cells are indicated by light blue arrows. Size bars are 50 microns.


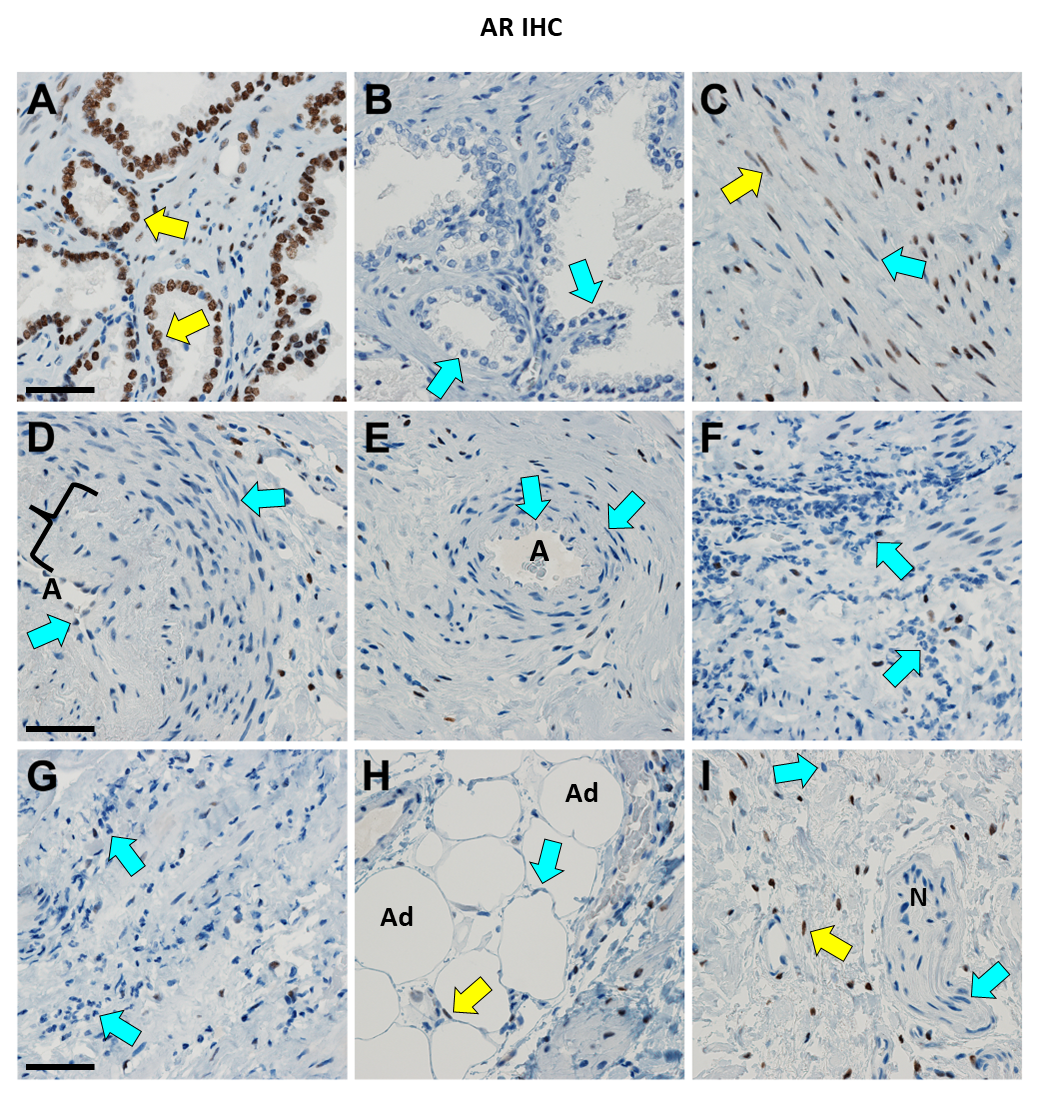


*Supplemental Fig 1d Androgen Receptor immunohistochemistry examples* AR IHC is shown for A) positive control normal prostate, B) normal prostate staining without use of the primary antibody (negative control), C) USL smooth muscle fascicle, D) large artery with NIH (bracket) showing tunica media, neointima cells and endothelial cells, E) artery (A) with 1 layer of tunica intima showing endothelial cells, F) neutrophils in connective tissue and between smooth muscle fascicles, G) neutrophils in connective tissue, H) adipocyte (Ad), and I) neural bundles with surrounding connective tissue. IHC positive cells are indicated by yellow arrows, IHC negative cells are indicated by light blue arrows. Size bars are 50 microns.
